# Supplementary material for: Ternary complex of Kif2A-bound tandem tubulin heterodimers represents a kinesin-13-mediated microtubule depolymerization reaction intermediate
Source: Nat Commun. 2018 Jul 6;9:2628. doi: 10.1038/s41467-018-05025-7 (PMC6035175; doi:10.1038/s41467-018-05025-7)
Supplement: Supplementary file 1 — Supplementary Information [file 41467_2018_5025_MOESM1_ESM.pdf]

Ternary complex of Kif2A-bound tandem tubulin heterodimers represents a kinesin-13-mediated microtubule depolymerization reaction intermediate

Trofimova et al.

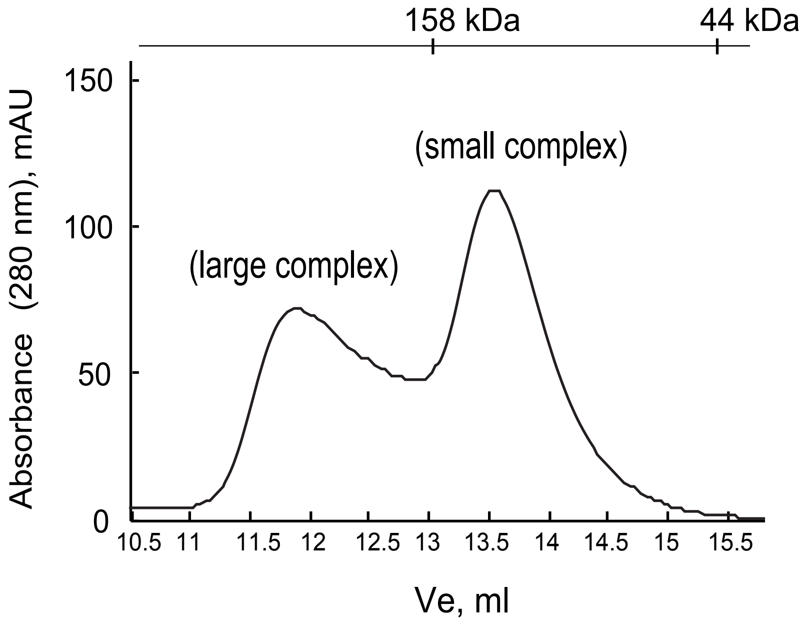

Supplementary Figure 1. Kif2A-NM-tubulin-DARPin small complex rearrenges to large complex upon concentration. Fractions corresponding to the small complex of Kif2A-NM-tubulin-DARPin werr concentrated 10-fold, and incubated for 30 min, and then re-run on the S200 10/300 GL column in HEPES buffer.

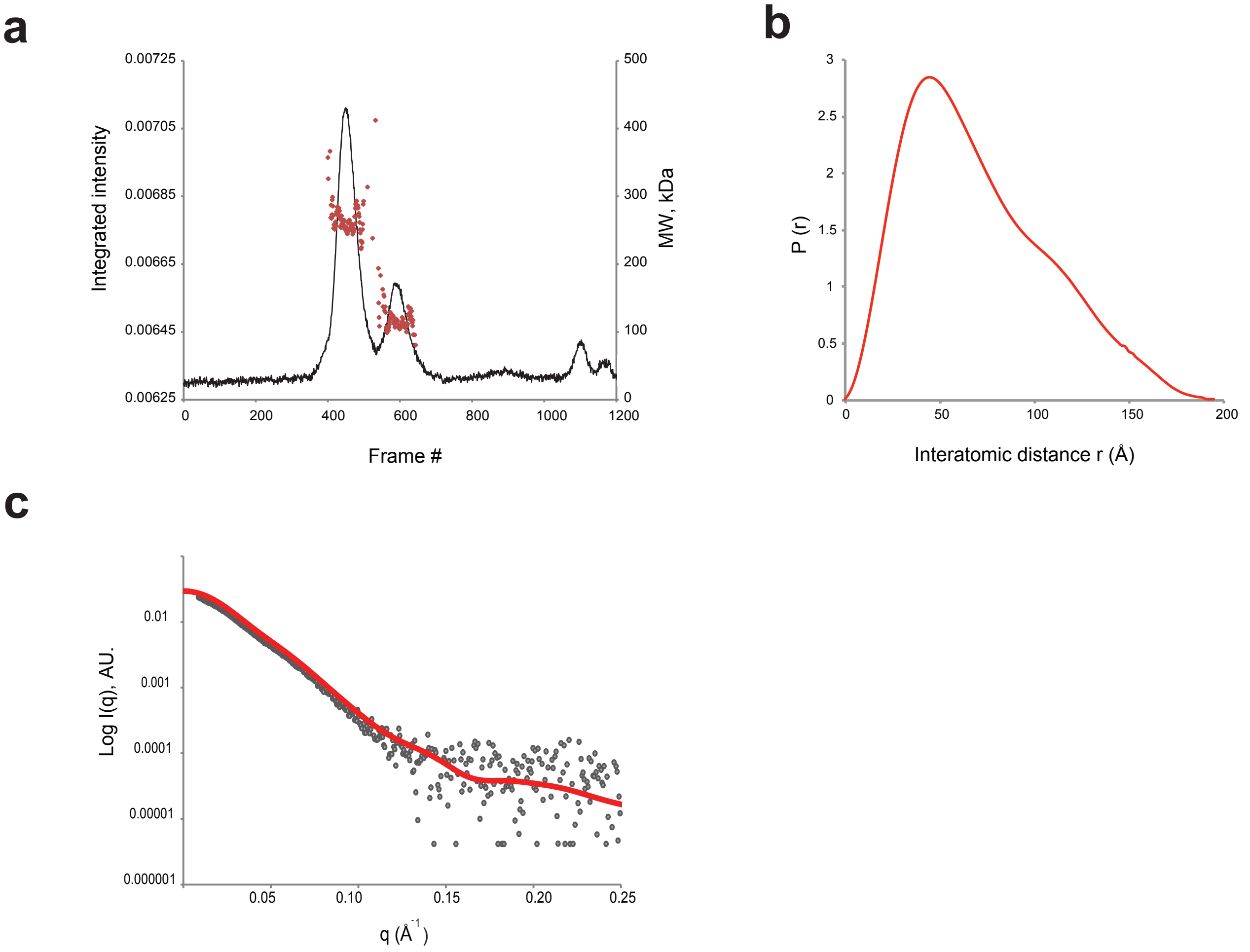

Supplementary Figure 2. SAXS analysis of the Kif2A-NM-tubulin-DARPin complex. (**a**) SEC-SAXS elution profile of Kif2A-NM-tubulin-DARPin mixture. Integrated intensity (black line) and the corresponding molecular weight correlations (red diamonds) were plotted across the elution peaks. (**b**) Pair-dis-tance (r) probability distribution computed from the experimental SAXS data for frames 433-473 by ATSAS. (**c**) CRYSOL comparison of the experimental scattering for the 1:2:1 Kif2A-NM-tubu-lin-DARPin complex (gray circles) with the simulated scattering proifle from the Kif2A-NM-tubulin-DARPin crystal structure (red line).

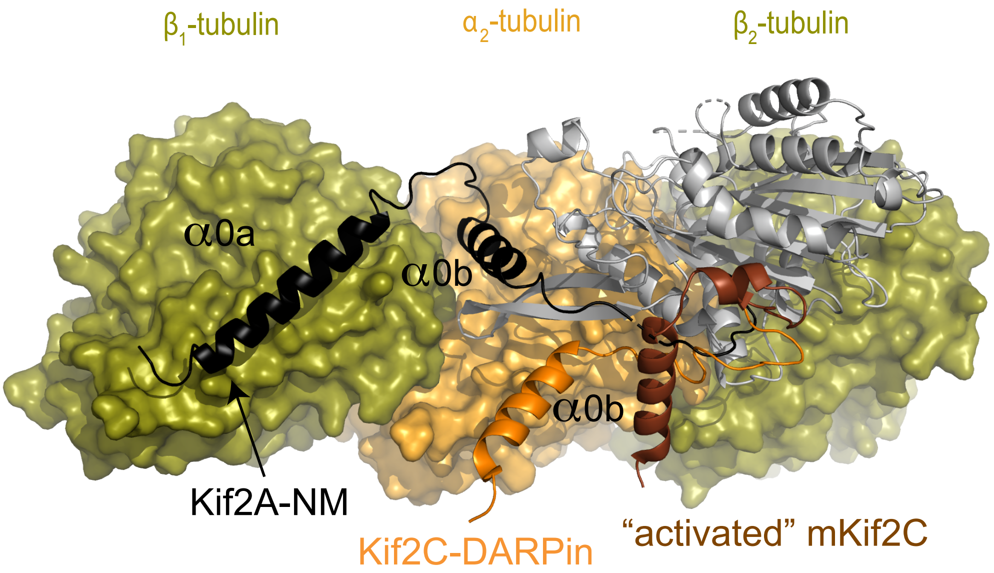

Supplementary Figure 3. Conformations of the neck helices of kinesin-13 proteins observed in crystal structures. Motor domain of Kif2A is represented in grey. The neck parts of Kif2A-NM-tubulin-DARPin complex, α0a and α0b, are in black. α0b of Kif2C-tubulin-DARPin complex (PDB ID: 5MIO) is in orange and α0b of the “activated conformation” of mKif2C (PDB ID: 5XJA) is in brown. Tubulin is shown as a surface representation.

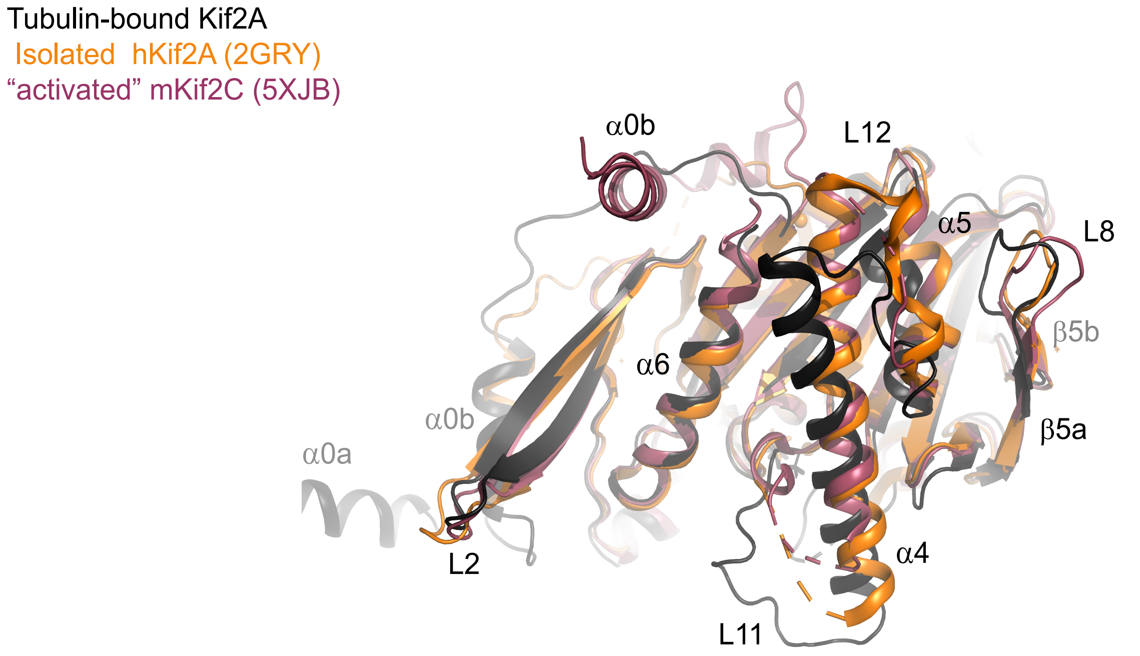

Supplementary Figure 4. Conformations of the Kif2A motor domain relative to “activated” Kif2C. The motor domain of tubulin-bound Kif2A-NM (black) is superimposed on isolated ADP-Kif2A (orange) and the mKif2Ccore:ADP-BeFx structure (maroon) via the P-loop. The r.m.s.d. for 94 equivalent Cα posi-tions in 5XJA is 3.63 Å, and the r.m.s.d. for 97 equivalent Cα positions in 5XJB is 3.59 Å). Proteins are viewed from tubulin surface.

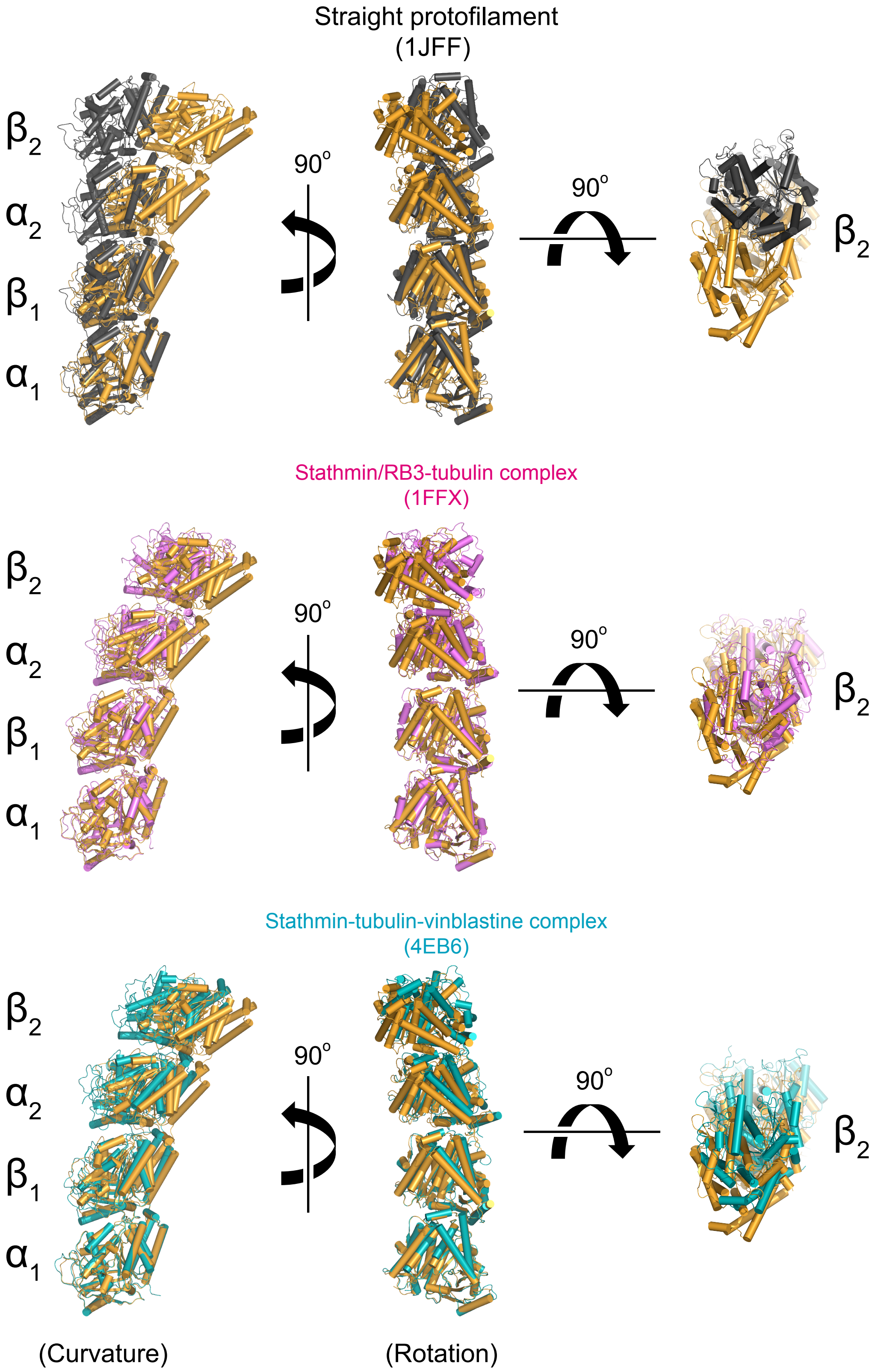

Supplementary Figure 5. Curvature and rotational displacement of tubulin complexes. Views are from the side (left), looking from the outer surface of the protofilament, into the lumenal
space (middle), and looking into the long axis of the protofilament from the plus-end (right). Supoerposition was performed using the α_1-tubulin subunit of each complex. Tubulin subunits of the Kif2A-NM-tubulin-DARPin complex are coloured orange.

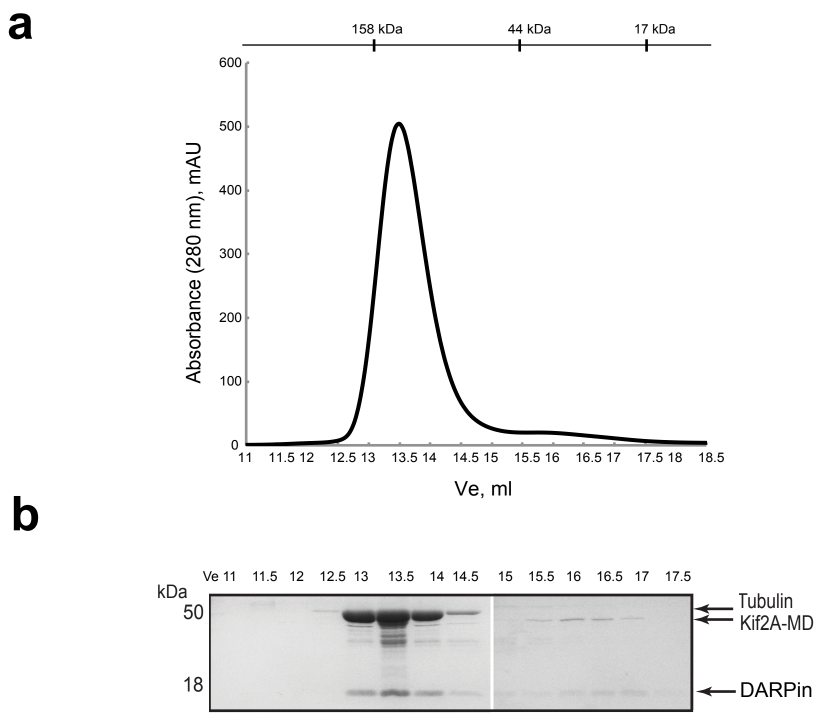

Supplementary Figure 6. Kif2A-MD forms a complex with tubulin in the presence of AMPPNP . (**a**) Size- exclution chromatography (SEC) profile of Kif2A-MD-tubulin-DARPin (1:1:1.05 molar ratio) supplemented with 1 mM AMPPNP in HEPES buffer. (**b**) 12% SDS-PAGE gel of SEC fractions from the above experiment. Molecular weight of Kif2A-MD = 42 kDa, tubulin = 50 kDa, and DARPin = 18 kDa

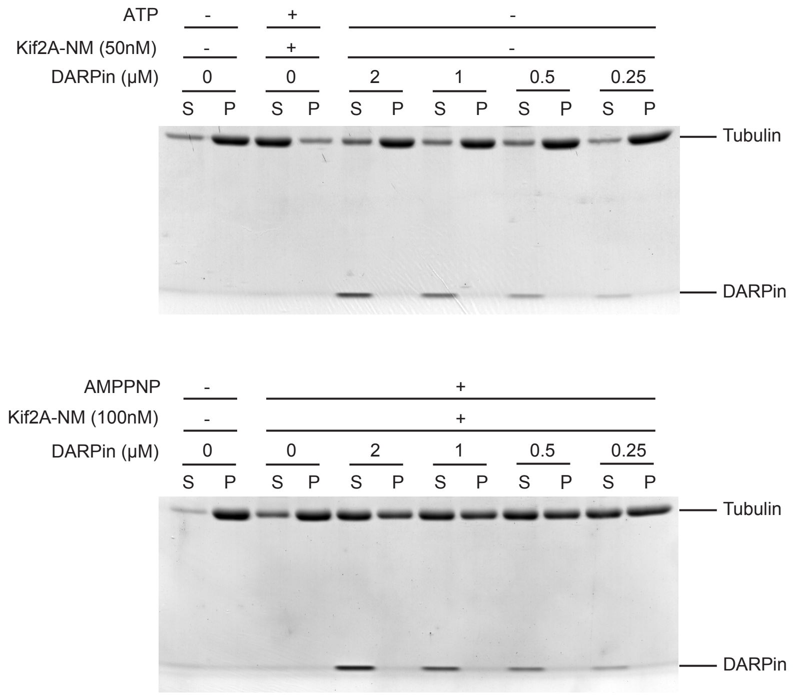

Supplementary Figure 7. Level of MT depolymerization in the presence of varying amounts of DARPin without (upper gel) or with Kif2A-NM (lower gel). The samples were processed as in Figure 7 and evaluated by MT sedimentation assay and coomassie blue stained gels.

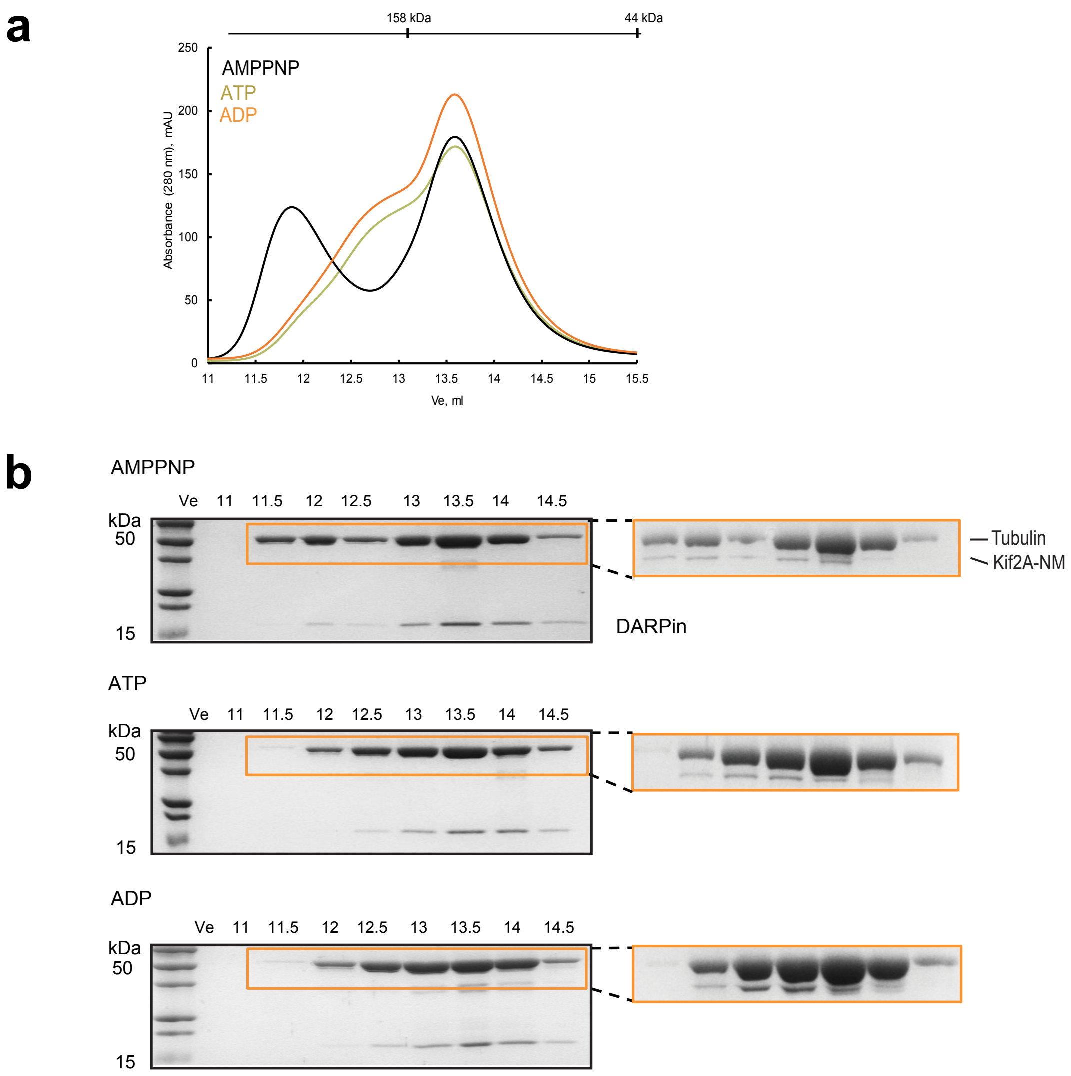

Supplementary Figure 8. Kif2A-NM-tubulin-DARPin complexes formed in the presence of different nucleotides. (**a**) Size-exclution chromatography (SEC) profiles of Kif2A-NM-tubulin-DARPin complexes (0.5:1:1.05 molar ratio) supplemented with 0.1 mM ATP (green), ADP (orange), and AMPPNP (black) separated on a S 200 10/300 GL column in HEPES buffer. (**b**)12% SDS-PAGE gels of SEC fractions from the above experiments. Molecular weight of Kif2A-NM = 48 kDa, tubulin = 50 kDa and DARPin = 18 kDa. Insets show Kif2A-NM-tubulin-DARPin fractions resolved on 10% SDS-PAGE gels.

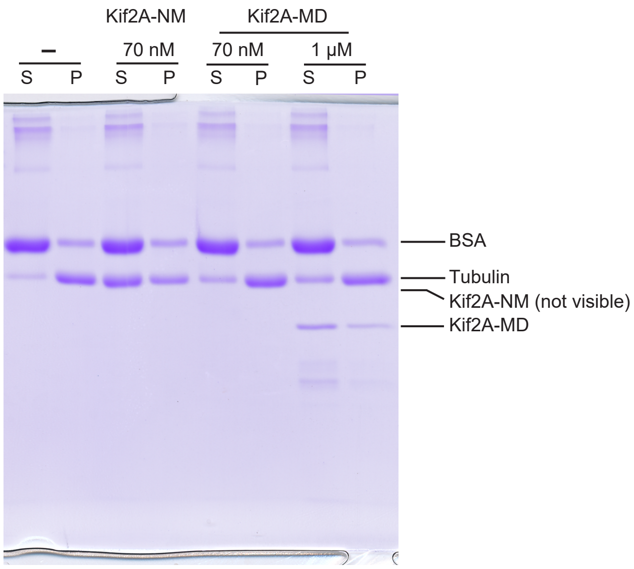

Supplementary Figure 9. Uncropped gel of Figure 1b

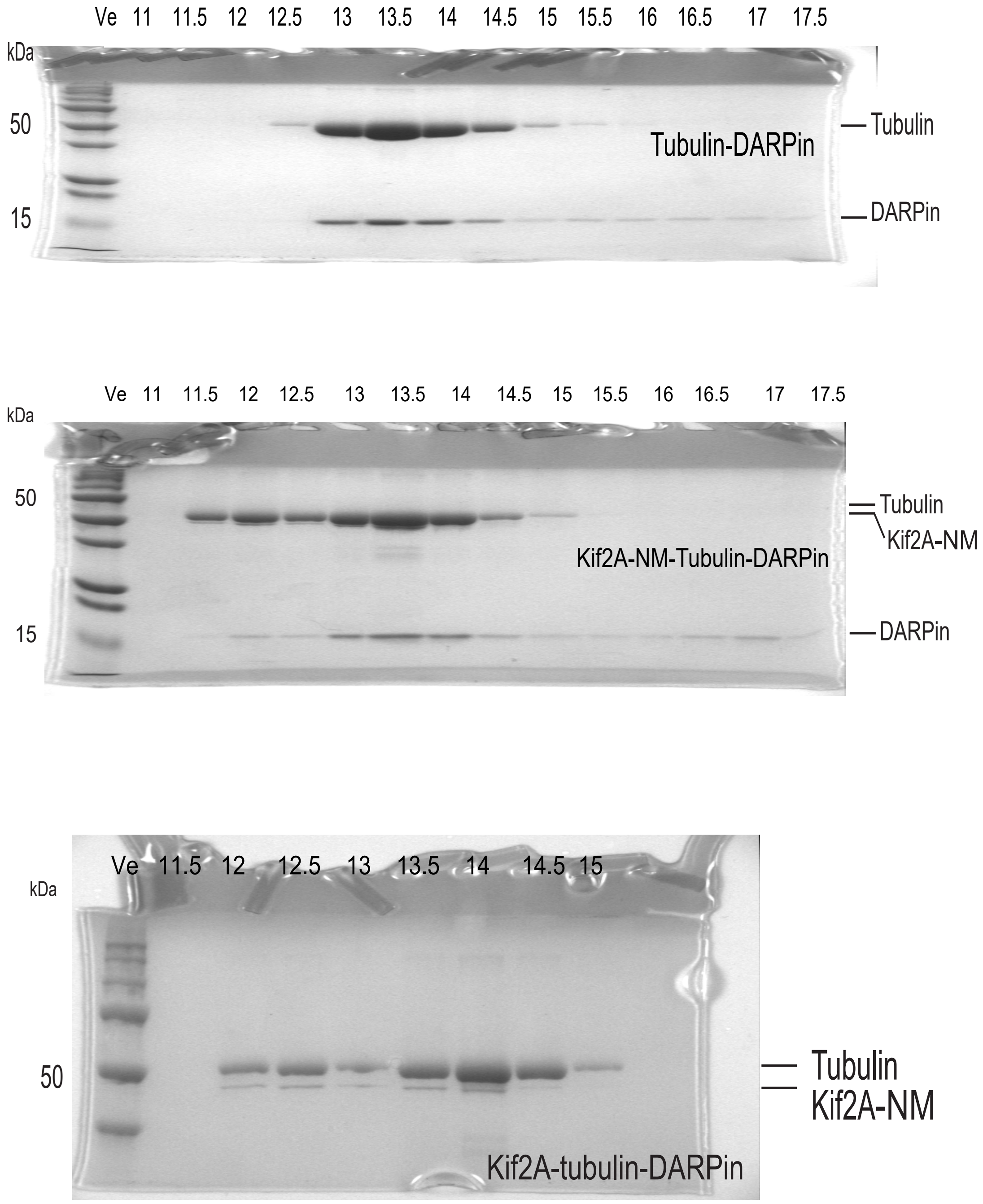

Supplementary Figure 10. Uncropped gels of Figure 1d

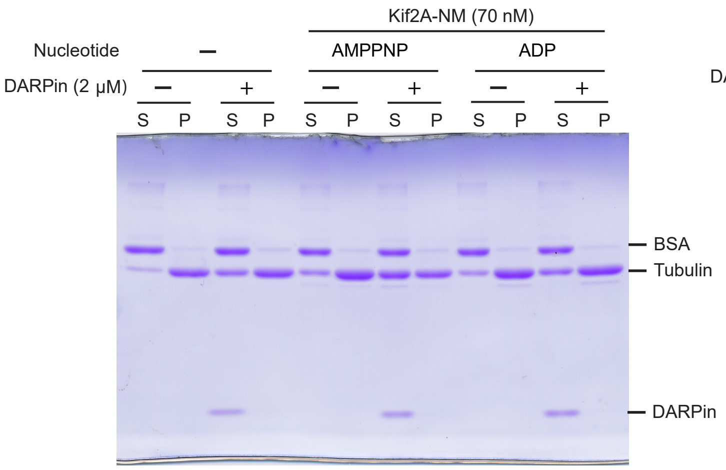

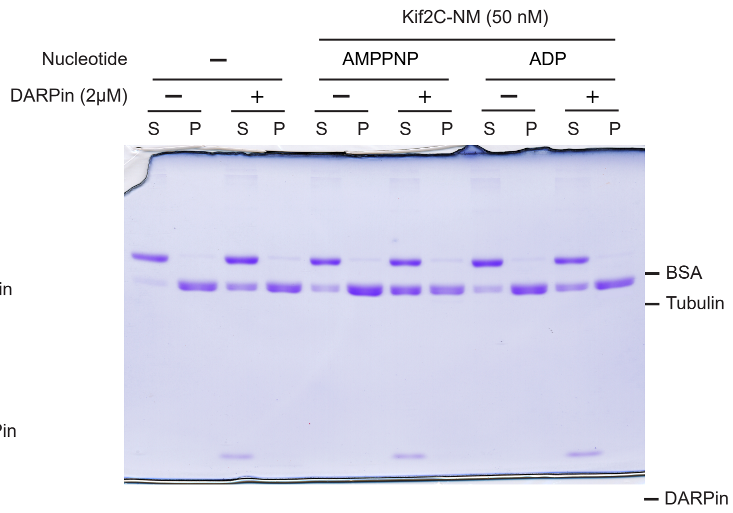

Supplementary Figure 11. Uncropped gels of Figure 7a and 7b

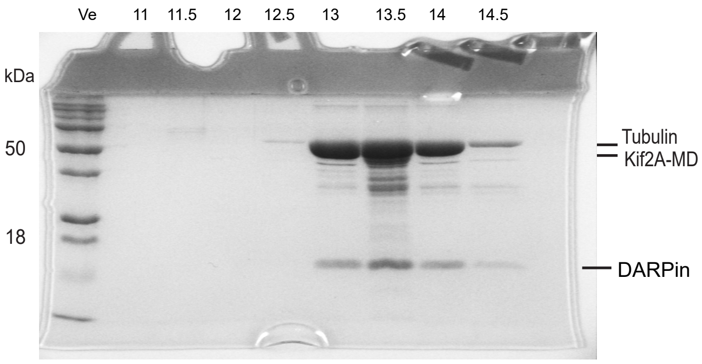

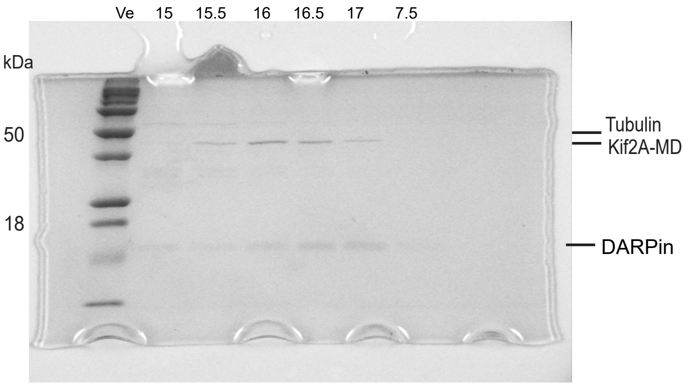

Supplementary Figure 12. Uncropped gels of Supplementary Figure 6

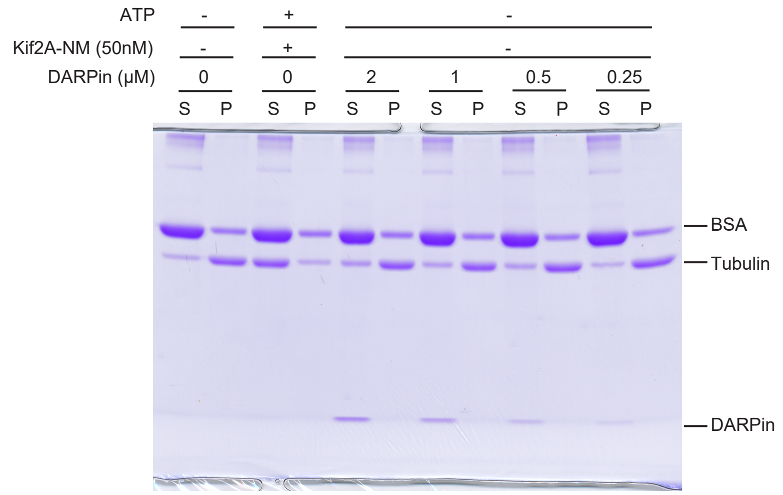

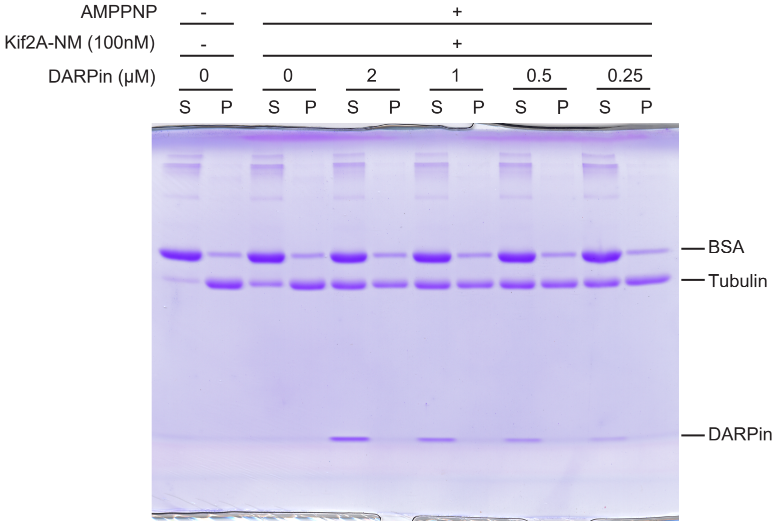

Supplementary Figure 13. Uncropped gels of Supplementary Figure 7

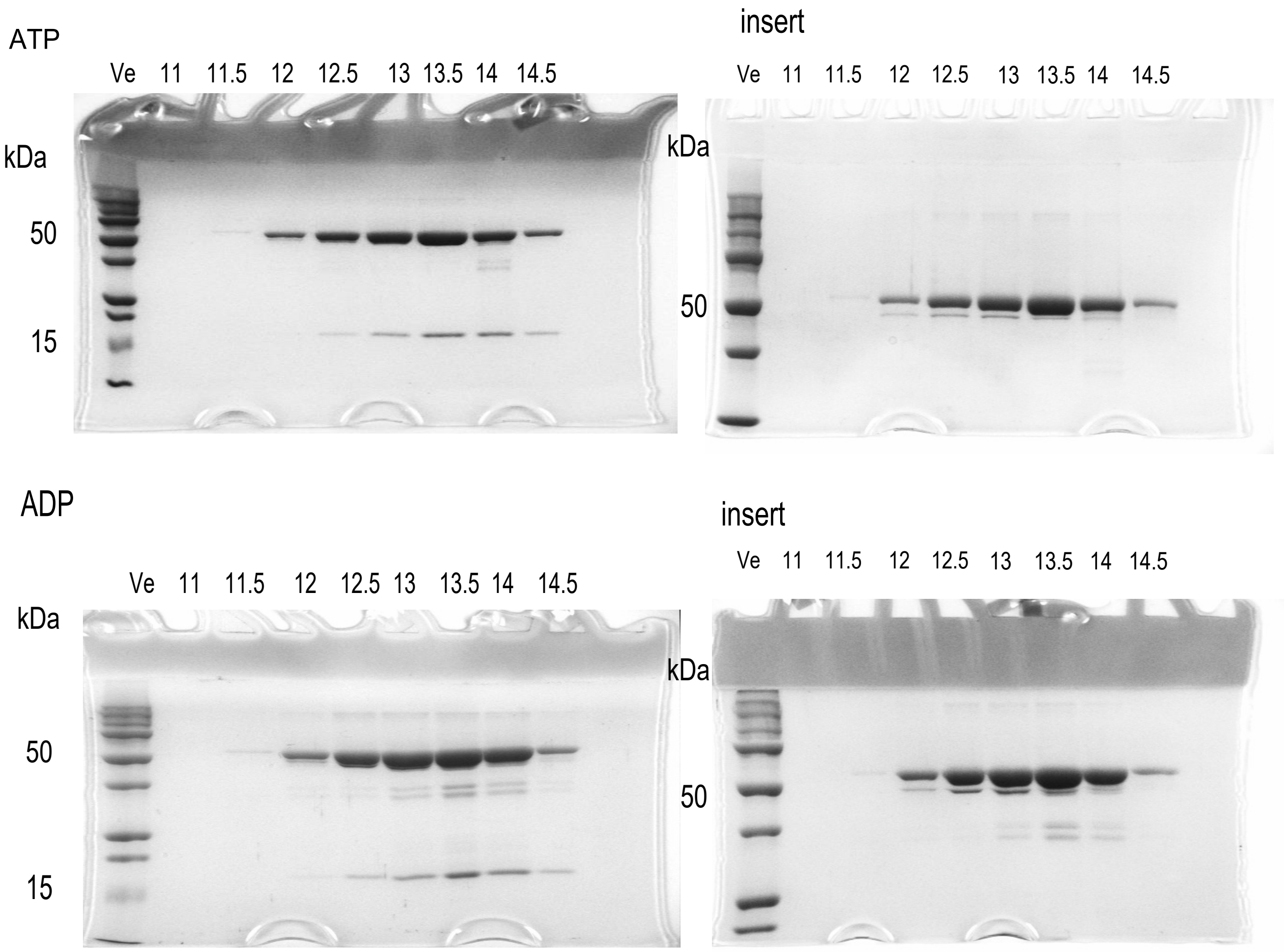

Supplementary Figure 14. Uncropped gels of Supplementary Figure 8b

Table S1. BioSAXS data collection and scattering-derived parameters.

Data collection parameters	
Beam line	G1 Station of the Cornell High Energy Synchrotron Source
Beam geometry	Beam size: 250 μm x 250 μm
Wavelength (Å)	1.267
Camera distance (mm)	1470
Exposure time (s)	2
Temperature (K)	295
Structural parameters	
I_0 (cm^{-1}) (from Guinier)	0.0298
R_g (Å) (from Guinier)	54.9
I_0 (cm^{-1}) (from P(r))	0.0298
R_g (Å) (from P(r))	55.9
D_{max} (Å)	195
Molecular mass determination	
Experimental Mw using a volume of correlation (kDa)	258.5
Calculated Mw from sequence (kDa)	266
Software employed	
Primary data processing	BioXTAS RAW
Ab initio analysis, validation and averaging	DAMMIF/DAMAVER
# of modeling iteration	10
χ^2 of ab initio model	0.991
DAMAVER NSD	0.74± 0.083
Computation of model intensities	CRYSOL
Comparison of theoretical profile with experimental data χ^2	1.093
Three dimensional graphics representations	Chimera
